# Similarity of *wh*-Phrases and Acceptability Variation in *wh*-Islands

**DOI:** 10.3389/fpsyg.2015.02048

**Published:** 2016-01-12

**Authors:** Emily Atkinson, Aaron Apple, Kyle Rawlins, Akira Omaki

**Affiliations:** Department of Cognitive Science, The Johns Hopkins UniversityBaltimore, MD, USA

**Keywords:** relativized minimality, *wh*-island, D-linking, acceptability judgment, amelioration, similarity interference

## Abstract

In *wh*-questions that form a syntactic dependency between the fronted *wh*-phrase and its thematic position, acceptability is severely degraded when the dependency crosses another *wh*-phrase. It is well known that the acceptability degradation in *wh*-island violation ameliorates in certain contexts, but the source of this variation remains poorly understood. In the syntax literature, an influential theory – Featural Relativized Minimality – has argued that the *wh*-island effect is modulated exclusively by the distinctness of morpho-syntactic features in the two *wh*-phrases, but psycholinguistic theories of memory encoding and retrieval mechanisms predict that semantic properties of *wh*-phrases should also contribute to *wh*-island amelioration. We report four acceptability judgment experiments that systematically investigate the role of morpho-syntactic and semantic features in *wh*-island violations. The results indicate that the distribution of *wh*-island amelioration is best explained by an account that incorporates the distinctness of morpho-syntactic features as well as the semantic denotation of the *wh*-phrases. We argue that an integration of syntactic theories and perspectives from psycholinguistics can enrich our understanding of acceptability variation in *wh*-dependencies.

## Introduction

Much work in syntax has investigated the acceptability of English sentences that involve multiple *wh*-phrases, as in (1):

(1) a. **Who** __ wondered **who** bought the car?
      b. ^∗^**What** did you wonder **who** bought __ ?

Despite the superficial resemblance of sentences in (1), native speakers of English perceive (1a) as a more acceptable sentence of English than (1b). This example illustrates the so-called *wh*-island constraint (Chomsky, 1964, 1977; cf. Ross, 1967): the grammar disallows dependency formation between the fronted *wh*-phrase (e.g., *what*) and its thematic position when there is another intervening *wh*-phrase (*who*). The discovery of this constraint raised a number of empirical and theoretical questions that remain unresolved: what types of representational or derivational constraints underlie the *wh*-island phenomenon? Are all *wh*-islands created equal, such that they all produce a similar degree of degradation? If not, what types of linguistic or cognitive factors affect the acceptability variation in *wh*-island violation?

The present paper aims to shed light on these questions through experimental tests of a recent, influential theory of *wh*-islands, called Featural Relativized Minimality (henceforth Featural RM; Friedmann et al., 2009; Belletti et al., 2012; Rizzi, 2013; for related proposals, see also Starke, 2001; Boeckx and Jeong, 2003). As the review below illustrates, there are two reasons why this theory deserves ample attention from syntacticians and psycholinguists. First, unlike many syntactic theories that only distinguish grammatical from ungrammatical sentences, Featural RM predicts fine variations in acceptability across different types of *wh*-islands, in particular, how the acceptability of *wh*-island violations can *ameliorate* depending on the similarity of *wh*-phrases. Second, as noted by Rizzi (2013), Featural RM resembles memory constraints on sentence processing, where the similarity of competing words in the sentence often predicts comprehension difficulties. As such, empirical investigations of *wh*-island amelioration effects provide a unique opportunity to explore the link between Featural RM and memory constraints in parsing. We report 4 experiments that explore the empirical predictions of Featural RM, and demonstrate that the theory needs refinement by incorporating aspects of memory encoding and retrieval constraints that guide the real-time computation of syntactic representations.

### Featural Relativized Minimality and Similarity Interference in Parsing

The definition of the Featural RM constraint can be summarized as in (2), which is slightly modified from Rizzi (2013) for expository purposes:

(2) In the configuration [… X … Z … Y …], X and Y cannot form a dependency if Z c-commands Y, and Z is the same structural type as X.

The syntactic condition as stated in (2) ensures that a *wh*-dependency cannot be established when there is a competing intervener [Z in (2)] that is structurally closer to the thematic position (Y) than the fronted *wh*-phrase (X). In Featural RM, the definition of the *structural type* that constitutes a violation of RM is stated in terms of morpho-syntactic features of those constituents.

A critical empirical observation that led to the use of morpho-syntactic features in Featural RM is the amelioration of *wh*-island violations with a D(iscourse)-linked *wh*-phrase (Pesetsky, 1987). While D-linked *wh*-phrases have been intuitively characterized as linked to previous discourse in some way, we will primarily use it here as a cover-term for *which*-phrases that denote a set of individuals. In the syntax literature, it has been reported that extracting the bare *wh*-phrase *what* from the *wh*-island, as in (3a), results in an ungrammatical sentence, but the extraction of the D-linked *wh*-phrase *which problem* in (3b) is considered marginally grammatical. This suggests that the *wh*-island violation in (3b) is somewhat ameliorated, though its acceptability is still degraded compared to the grammatical *wh*-extraction in (3c).

(3) a. ^∗^**What** do you wonder **who** solved **__**?
      b. ?**Which problem** do you wonder **who** solved **__**?
      c. **Which problem** do you think that John solved **__**?

Assuming the acceptability pattern indicated in (3), Rizzi and colleagues proposed that the degree of overlap in morpho-syntactic features of *wh*-phrases accounts for the acceptability variation (Friedmann et al., 2009; Belletti et al., 2012; Rizzi, 2013). For example, the feature relation between the two *wh*-phrases can be characterized as identity (3a), inclusion (3b), and disjunction (3c). In (3a), the extracted constituent and the intervener both contain only a [+Q(uestion)] feature, and hence the feature sets are identical. This *identity* relation results in a severe degradation in acceptability. In (3b), the intervener only contains [+Q], whereas the feature set for the D-linked *wh*-phrase contains [+Q] as well as [+N(oun)], the latter of which represents the “referential status” of the D-linked *wh*-phrase (see Cinque, 1990). This configuration is called an *inclusion* configuration, as the extracted constituent is more richly specified, and its feature set is a superset of that of the intervener. This inclusion relation leads to a less severe degradation in acceptability, and the *wh*-island effect is ameliorated relative to (3a), but the sentence is not necessarily judged as fully acceptable. Finally, in (3c) the embedded clause contains no [+Q] feature, and hence the feature specifications for the extracted constituent and the (potential) intervener are distinct. This is termed a *disjunction* configuration, which leads to no violation of Featural RM. These three feature set relations and their well-formedness statuses are summarized in **Table 1**.

**Table 1:** Taxonomy of feature set and well-formedness in Featural RM.

X *Fronted phrase*	Z *Intervener*	Y *Thematic position*	Well-formedness	Type
+A	+A	<+A>	Ungrammatical (^∗^)	Identity
+A, +B	+A	<+A, +B>	Marginal (?)	Inclusion
+A	+B	<+A>	Grammatical (√)	Disjunction

In summary, a key property of Featural RM is that it is concerned with the similarity of the fronted constituent and intervener in terms of morpho-syntactic features: the overlap of features causes degradation, and amelioration is observed when the extracted constituent has a richer or distinct set of morpho-syntactic features than the intervener.

The data discussed above concern the acceptability of sentences, but related observations have been made in adult and child sentence processing research on comprehension of filler-gap dependencies. For example, children experience greater comprehension difficulties with object *wh*-questions like *Which dog did the cat bite __ ?* than *Who did the cat bite __ ?*, possibly due to the overlap of [+N] feature in the fronted *wh*-phrase *which dog* and the intervening NP *the cat* (Friedmann et al., 2009; Belletti et al., 2012; for counter-arguments, see Goodluck, 2010; Bentea and Durrleman, 2014). In adult sentence processing, object relative clauses with two definite Noun Phrases (NPs) like *The banker that the barber praised* __ pose greater comprehension difficulties than sentences in which the intervening NP is replaced by a pronoun or a name, as in *The banker that you/John praised__* (Gordon et al., 2001, 2002, 2004, 2006; Warren and Gibson, 2002, 2005). This adult finding may be compatible with Featural RM if we expand the relevant morpho-syntactic features to include features that distinguish definite NPs from pronouns or names.

An alternative explanation, which has received much support from sentence processing as well as domain-general working memory research, is that these observations reflect constraints on memory encoding and retrieval mechanisms, which are subject to so called *similarity-based interference* (Lewis and Vasishth, 2005; for a review, see Van Dyke and Johns, 2012). There are two ways in which similarity-based interference could occur. The first and more well-known type of similarity-based interference is *retrieval interference*. Comprehension of relative clauses or *wh*-questions requires the parser to retrieve the fronted *wh*-phrase and relate it to its thematic position. According to these memory accounts, this retrieval mechanism uses a cue-based search process, and activates all NPs that meet (some of) the search cues. The retrieval competition among candidates with similar features results in comprehension difficulties. The second type is called *encoding interference*. This type of interference is observed when the parser encounters words or phrases that are similar to one another, and the process of encoding and storing them as distinct items in memory is disrupted. The resulting representations that are stored in memory may be less precise or robust, and may require more cognitive resources to retrieve later in the sentence (see Gordon et al., 2002).

This raises questions about whether the variation of acceptability judgments in (3) may also be an instance of similarity-based interference: the identity relation in (3a) causes greater similarity-based interference than the inclusion configuration in (3b), which in turn causes more interference than (3c). In fact, it may even be possible to reduce Featural RM (**Table 1**) to constraints on working memory. However, as noted by Rizzi (2013), one key difference between Featural RM and memory retrieval accounts is that Featural RM is strictly concerned with the overlap of morpho-syntactic features, whereas similarity-based interference is typically sensitive to a variety of similarities, including semantic features (Van Dyke and McElree, 2006; Hofmeister, 2011; Hofmeister and Vasishth, 2014; Kush et al., 2015). Thus, further investigations of the role of semantic overlap in *wh*-island amelioration could shed light on the link between Featural RM and similarity-based interference.

### The Present Study

The present study uses acceptability judgment experiments to explore the role of morpho-syntactic and semantic features in amelioration of *wh*-island violations. Specifically, we will explore the acceptability of the inclusion configuration (4a), and how it compares to the acceptability of the D-linked identity configuration (4b).^1^

(4) a. **Which athlete** did she wonder **who** would recruit __? (Inclusion)
      b. **Which athlete** did she wonder **which coach** would recruit __? (D-linked identity)

In (4a) the extracted *wh*-phrase is D-linked and the intervener is a bare *wh*-phrase, whereas in (4b), both the extracted *wh*-phrase and the intervener *wh*-phrase are D-linked. Under Featural RM, the dependency in (4b) should be classified as an identity configuration, since both *wh*-phrases have features [+Q, +N]. We will refer to this configuration as *D-linked identity*, to distinguish it from the typical identity configuration [e.g., (3a)] that only includes bare *wh*-phrases. The dependency in (4a) is an inclusion configuration, since the intervening *wh*-phrase only has the feature [+Q]. Given these assumptions about the morpho-syntactic features, Featural RM predicts that (4b) should be less acceptable than (4a). On the other hand, both *wh*-phrases in the D-linked identity configuration (4b) are semantically more specific, as they characterize distinct sets of individuals: a set of athletes and a set of coaches. The *wh*-phrases in (4a) are less distinct because they do not denote distinct sets: the set of athletes is a proper subset of the set of people denoted by *who*. Thus, if semantic distinctness plays a role in dependency formation, the D-linked identity configuration (4b) may cause less similarity-based interference and lead to *wh*-island amelioration, possibly more so than in the inclusion condition (4a).

Informal judgment data reported in the syntax literature (Pesetsky, 1987, 2000; Comorovski, 1996; Shields, 2008) suggest that the D-linked configuration in (4b) should be more acceptable than the inclusion configuration in (4a); in fact, Pesetsky originally annotated them as fully grammatical, in contrast to non-D-linked identity examples. This may challenge the predictions of Featural RM, but it may reflect the fact that differences such as (4a) vs. (4b) are extremely subtle, and the reliability of the data in (4) may be in question. Although D-linked *wh*-phrases are reported to ameliorate *wh*-island violations, those sentences are still often described as unacceptable or ungrammatical to some degree. In other words, sentences like (4a) differ from non-D-linked identity sentences only in the severity of degradation, which is not guaranteed to be readily distinguishable in informal judgments. While D-linked identity examples are often (but not uniformly) annotated as fully grammatical in the linguistics literature, there is evidence that they have a different status than non-D-linked identity examples (Pesetsky, 2000; Shields, 2008). For example, Pesetsky (2000) demonstrates that they, unlike regular grammatical multiple-*wh* examples, e.g., (1a), show intervention effects, e.g., ^∗^*Which book didn’t which person read?* Because the contrasts are empirically subtle and complex, we will use acceptability judgment experiments with a 7-point scale that provide a quantitative measure of acceptability variation. Such experiments have proven useful for a variety of syntactic phenomena that involve subtle contrasts in acceptability intuitions (e.g., McDaniel and Cowart, 1999; Featherston, 2005; Alexopoulou and Keller, 2007; Hofmeister and Sag, 2010; Sprouse et al., 2012; Sprouse and Hornstein, 2013).

In fact, several experimental studies have provided preliminary evidence that semantic information may indeed play a role in island amelioration (Alexopoulou and Keller, 2013; Goodall, 2015; see also Fanselow et al., 2011). Alexopoulou and Keller (2013) investigated the acceptability of extraction out of *whether*-islands (e.g., *What does Claire wonder whether we will watch __ at the cinema?*) while manipulating the animacy and D-linking status of the *wh*-phrase (e.g., *what, who, which movie, which colleague*). Here, it was found that bare inanimate *wh*-phrase *what* was less acceptable than the other three *wh*-phrase types, which did not differ from each other. This may suggest that inanimate nouns may be easier to extract out of an island, but this result is difficult to relate to the present study for two reasons. First, the animacy effect did not hold for the D-linked *wh*-phrases, suggesting that this may not be a robust effect. Second, *whether*-islands are different from *wh*-islands in (4) since the intervener (i.e., *whether*) itself does not relate to another (distant) thematic position. Goodall (2015) found clear evidence that D-linked *wh*-phrases ameliorate *wh*-islands that are more similar to those used in the present study. However, his D-linking manipulation compared bare *wh*-phrase against partitive *wh*-phrase (*What / Which of the cars do you wonder who might buy __ ?*). We note that, potentially, this partitive *wh*-phrase may have inflated the amelioration effect for a variety of reasons; for example, it contains a richer semantic content, which is known to facilitate retrieval processes in general (Hofmeister, 2011; Hofmeister and Vasishth, 2014). For this reason, our experiments will focus on D-linking manipulation that does not involve the partitive, in line with the D-linking manipulation that has been used more widely in the syntax literature.

Before presenting the experiments, it is important to clarify the scope of the present paper. The similarity-based interference accounts provide the motivation for the present study, as well as the critical predictions that semantic similarity should also play a role in acceptability variation in *wh*-islands. However, oﬄine acceptability judgment data that we report here does not necessarily shed light on whether the observed acceptability variation in *wh*-islands actually reflects working memory constraints on encoding and retrieval processes during real-time sentence processing. As such, our aim is not to investigate how acceptability variation unfolds during real-time sentence processing, but rather to test whether the ultimate acceptability judgment data is compatible with the predictions of the similarity-based interference accounts.^2^

## Experiment 1

This experiment investigates the acceptability of *wh*-island violations with D-linked identity and *wh*-island violations with an inclusion configuration, where only the extracted phrase is D-linked. We test this using a 2 × 2 design with movement from within a *wh*-island (non-island vs. island) and feature relation (non-identity vs. identity) as factors, as in **Table 2**. The extraction conditions contain extractions out of *wh*-islands. The non-extraction counterparts in do not contain *wh*-island violations and, hence, serve as baseline conditions.

**Table 2:** Sample item set from Experiment 1.

Non-identity	Non-island	Which student __ wondered who would invite the visitor?
	Island	Which visitor did you wonder who would invite __?
			*(Inclusion)*

Identity	Non-island	Which student __ wondered which teacher would invite the visitor?
	Island	Which visitor did you wonder which teacher would invite __?
			*(D-linked Identity)*

Featural RM predicts that the D-linked identity condition should be severely degraded because the set of features on both D-linked *wh*-phrases (*which NP*, [+Q, +N]) are identical. On the other hand, the inclusion configuration should be less degraded than D-linked identity, because the features on the fronted phrase (*which NP*, [+Q, +N]) are a superset of the features on the intervener (*who*, [+Q]).

### Method

#### Participants

Twenty-five self-reported native English speakers were recruited on the internet via Amazon Mechanical Turk, which has proven to be a useful venue in which participants provide reliable acceptability judgment data (Gibson et al., 2011; Sprouse, 2011). They were paid $0.30 for their participation. The data from 3 additional participants was excluded from the analysis, as they only used the extreme ends of the scale in the pre-test phase (see below). This and the following experiments were approved by the Johns Hopkins University Institutional Review Board, and all participants provided informed consent.

#### Materials

The stimuli for this experiment consisted of 16 sets of bi-clausal *wh*-questions (**Table 2**). These 16 items were counter-balanced across four lists, so that each participant saw only one version of each target item. Forty-eight filler items of comparable length and varying acceptability were randomly interspersed with these target items for a total of 64 items. Based on our informal judgments and acceptability judgment data in the literature, we manipulated the acceptability of filler items to create three groups of fillers: those that are expected to receive high acceptability rating (good fillers), those that are expected to receive low rating (bad fillers), and sentences whose acceptability was expected to fall in between (middle fillers). Fillers consisted of both declaratives and questions, which were included to ensure that the target items were not the only questions in the experiments. Having filler items with varying acceptability serves two purposes. First, this encourages the participants to use a large portion of the scale, which is critical for revealing subtle contrasts. Second, the data from fillers can serve as a baseline measure that can be used to estimate the magnitude of amelioration effects in target sentences. Stimuli from all four experiments, including the fillers, are provided in Supplementary Materials.

#### Procedure

All of the acceptability judgment experiments in this paper have the same basic procedure. Participants were instructed to rate sentences on a scale from 1 (bad) to 7 (good). Before beginning the experiment, participants were provided with detailed instructions and examples to illustrate that the task is not about stylistic considerations, prescriptive norms, or the plausibility of the event described. This was followed by additional examples with varying degrees of acceptability to illustrate what type of sentence corresponded to different parts of the scale. None of these example sentences used the same structure as the target sentences shown in (5).

Additionally, the first six experimental trials were identical for all participants and served as a pre-test phase. These six trials consisted of two highly acceptable sentences, two highly unacceptable sentences, and two marginal ones. These sentences were included to encourage participants to use the entire scale. The use of a large range of points on the scale was critical for the present study, because the target comparison involves two unacceptable sentence conditions. The acceptability contrast between such sentences may not be revealed if participants used, for example, only the two extreme ends of the scale and treated the task as a binary judgment task. If participants restricted their judgments to the extreme ends of the scale (i.e., 1 and 7) on these initial items, the data from these participants were excluded from further analyses, as it suggests that the participants are treating the scale as if it is a binary choice, which may skew the acceptability ratings in unexpected ways.^3^

#### Data Analysis

All experiments in this paper use the same data analysis procedure. First, the raw judgment ratings, including both targets and fillers, were converted to *z*-scores within participants (Schütze and Sprouse, 2013). The *z*-score transformation converts a participant’s scores to units that represent the number of standard deviations a particular rating is from that participant’s mean rating. This procedure corrects for the potential that individual participants treat the scale differently, e.g., using only a subset of the available ratings, because it standardizes all participants’ results to the same scale. We also ran the reported analyses with the raw ratings and the results were unchanged in all experiments, although we will only report data and analyses based on *z*-scores.

Linear mixed-effect models were used to analyze the data; these models allow the simultaneous inclusion of random participant and random item variables (Baayen et al., 2008). Each model was fit using the maximal random effects structure that converged (Barr et al., 2013). These models were run in the R environment (R Core Development Team, 2015) using the lme4 package (Bates et al., 2015). *P*-value estimates for the fixed and random effects were calculated using the Sattherwaite approximation in the lmerTest package (Kuznetsova et al., 2015). When the results showed a significant interaction, planned pairwise comparisons were also performed to determine significance between individual conditions. These pairwise comparisons used separate linear mixed-effects models with maximal random effects structure; unlike other statistical analysis methods, mixed-effects models are robust to multiple comparisons.

### Results

**FIGURE 1:**
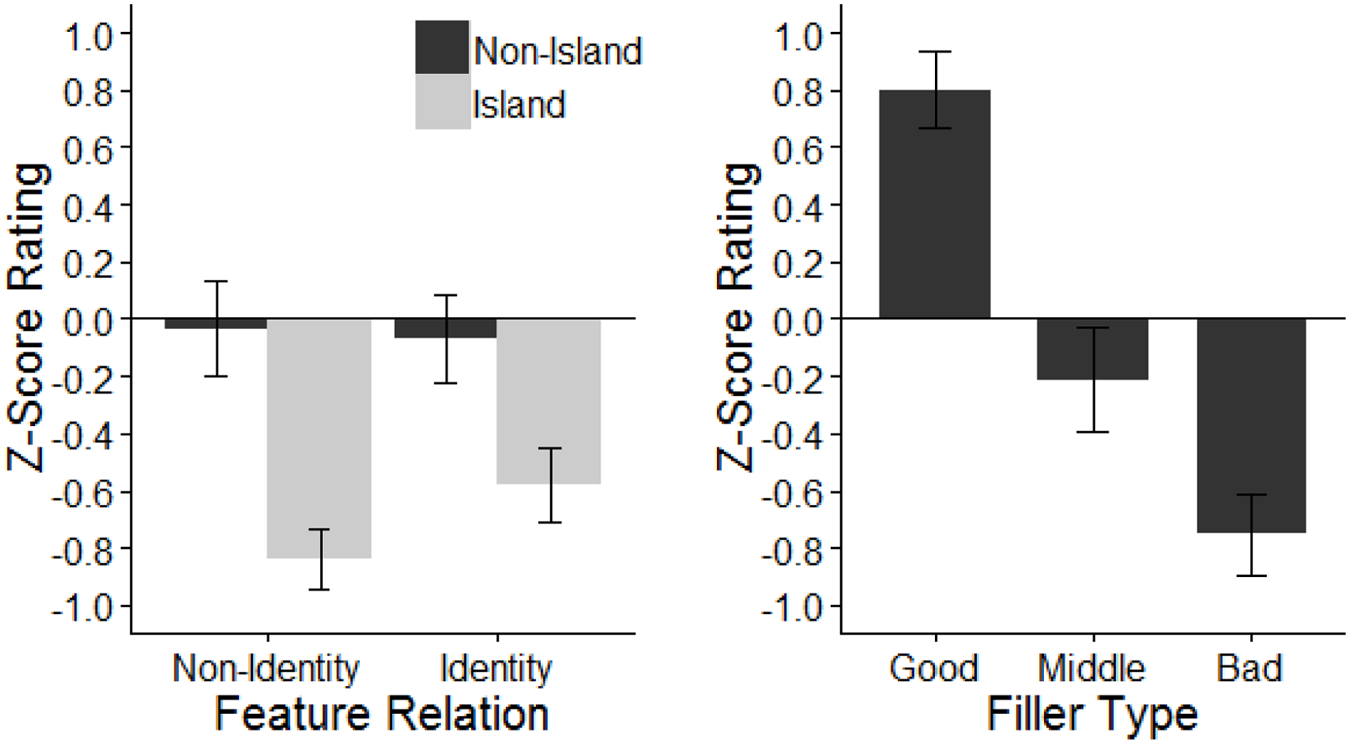
**Mean *z*-score acceptability rating of target questions by *wh*-phrase combination and islandhood, and mean *z*-score acceptability rating of filler sentences by filler type.** Error bars indicate ± 1 SE.

**Figure 1** presents the *z*-score transformed average ratings for each condition and for each filler type. Good filler sentences were rated as most acceptable (mean *z*-score = 0.80), while bad fillers were rated as least acceptable (mean *z*-score = -0.75). Middle fillers received ratings near participants’ mean rating (i.e., near a *z*-score of 0, mean = -0.21). This pattern of acceptability for the fillers is common across all four experiments.

For the target items, we found that the island conditions were rated as less acceptable than the non-island conditions (island mean *z*-score = -0.71, non-island mean *z*-score = -0.05). Within the island conditions, the D-linked identity condition is rated as more acceptable than the inclusion condition (-0.58 vs. -0.84). In the non-island conditions, average *z*-scored ratings are around zero (means -0.04 and -0.07), suggesting that they were rated close to individual participants’ mean ratings. This likely reflects the fact that sentences with two *wh*-phrases are generally uncommon and difficult to process out of context.

**Table 3** presents the estimated coefficients and the standard error for the Linear Mixed Effect model with islandhood and feature relation as fixed effects and random intercepts and slopes for participants and items. Significant effects are marked by their beta estimates.

**Table 3:** Fixed effects summary for Experiment 1 with maximal by-participant and by-item random effects.

	Estimate	*SE*
Intercept	-0.38^∗∗∗^	0.08
Islandhood	-0.66^∗∗∗^	0.11
Feature relation	-0.03	0.10
Islandhood × Feature relation	0.28^∗^	0.13

*^∗^p ≤ 0.05, ^∗∗^p ≤ 0.01, ^∗∗∗^p ≤ 0.001.*

There is a main effect of islandhood such that *wh*-island violations are significantly less acceptable than non-island violating questions. There is no main effect of feature relation, but there is a significant interaction of islandhood and feature relation. The estimated coefficient of this interaction indicates that the feature combination had a significant effect in the island conditions, but not in the non-island conditions. This is supported by planned pairwise comparisons: the two non-island conditions are not significantly different from one another (β = -0.02, *SE* = 0.12, *p* > 0.1), while the D-linked identity condition is rated as significantly more acceptable than the inclusion condition (β = 0.26, *SE* = 0.09, *p* < 0.01).

### Discussion

The results indicate that movement out of a *wh*-island generally results in severe degradation of acceptability. More importantly, this degradation is modulated by the feature relation between the two *wh*-phrases: the D-linked identity condition shows greater acceptability than the D-linked inclusion condition. These results replicate informal acceptability judgments in the literature that D-linking ameliorates *wh*-island effects, as well as judgment contrasts that D-linked identity leads to greater acceptability than inclusion (Comorovski, 1996; Shields, 2008). However, these results are not easily explained by the current formulation of Featural RM, which predicted that an identity configuration should be more degraded than an inclusion configuration. In fact, our results indicate that the D-linked identity configuration leads to a greater amelioration of the *wh*-island violation than an inclusion configuration.

We have so far focused only on the D-linked identity configuration. No items in this first experiment involve an identity configuration with bare *wh*-phrases, even though Rizzi’s (2013) proposal critically relies on an acceptability difference between an identity configuration with bare *wh*-phrases and an inclusion configuration with a fronted, D-linked *wh*-phrase. In order to confirm the presence of *wh*-island amelioration in the inclusion configuration, as predicted by Featural RM, Experiment 2 compares the inclusion condition against a D-linked identity condition as well as a bare identity condition, where both the fronted *wh*-phrase and the intervener are bare *wh*-phrases.

## Experiment 2

### Method

#### Participants

Thirty-two self-reported native English speakers participated via Amazon Mechanical Turk. They were paid $0.50 for participating.

#### Materials

The stimuli for this experiment consisted of 24 sets of biclausal sentences, which were constructed by using a 2 × 2 × 2 design with three factors: matrix *wh*-phrase (bare vs. D-linked), feature relation (non-identity vs. identity), and islandhood (non-island vs. island). The experimental conditions shown in **Table 4** include the same four conditions as Experiment 1 (those with a D-linked matrix *wh*-phrase) as well as four new conditions (those with a bare matrix *wh*-phrase) to test Featural RM’s broader predictions for *wh*-island amelioration effects. First, the acceptability of the island conditions is predicted to be significantly lower than that of non-island conditions. Second, Featural RM predicts that the identity island conditions should be the most severely degraded compared to all other conditions, including their non-island counterparts. It also predicts that the magnitude of degradation should not differ between the two identity island conditions. Third, the inclusion configuration should yield an amelioration of *wh*-island violations. Thus, the inclusion condition should yield a degradation compared to its non-island counterpart due to a *wh*-island violation, but the resulting acceptability should still be higher than the island identity conditions. Finally, the reverse inclusion configuration and its non-island counterpart are included in the design to test all combinations of the three factors we used in this experiment. The feature set taxonomy of Featural RM (see **Table 1**) does not make explicit predictions for these conditions; however, given that Rizzi and colleagues generally attribute the amelioration effects to the superset-subset relation of feature set between the fronted *wh*-phrase and intervener, we can infer the predictions of Featural RM to be that the acceptability of the reverse inclusion configuration should be similar to that of the two island identity conditions, and lower than the acceptability of the inclusion condition.

**Table 4:** Sample item set from Experiment 2.

Bare matrix *wh*-phrase	Non-identity	Non-island	Who __ wondered which teacher would invite the visitor?
		Island	Who did you wonder which teacher would invite __?
			*(Reverse Inclusion)*
	Identity	Non-island	Who __ wondered who would invite the visitor?
		Island	Who did you wonder who would invite __?
			*(Bare Identity)*

D-linked matrix *wh*-phrase	Non-identity	Non-island	Which student __ wondered who would invite the visitor?
		Island	Which visitor did you wonder who would invite __?
			*(Inclusion)*
	Identity	Non-island	Which student __ wondered which teacher would invite the visitor?
		Island	Which visitor did you wonder which teacher would invite __?
			*(D-linked Identity)*

These 24 items were counter-balanced across eight lists, so that each participant saw only one version of a target item. Forty-eight filler items of comparable length and varying acceptability were randomly interspersed with these target items.

#### Procedure and Data Analysis

This experiment used the same procedure and data analysis steps as Experiment 1. In the statistical analysis, we added planned pairwise comparisons for the island version of the bare identity, inclusion, and D-linked identity conditions, as the comparison of these three conditions is critical for establishing the amelioration of *wh*-island violations that are predicted by Featural RM.

### Results

**FIGURE 2:**
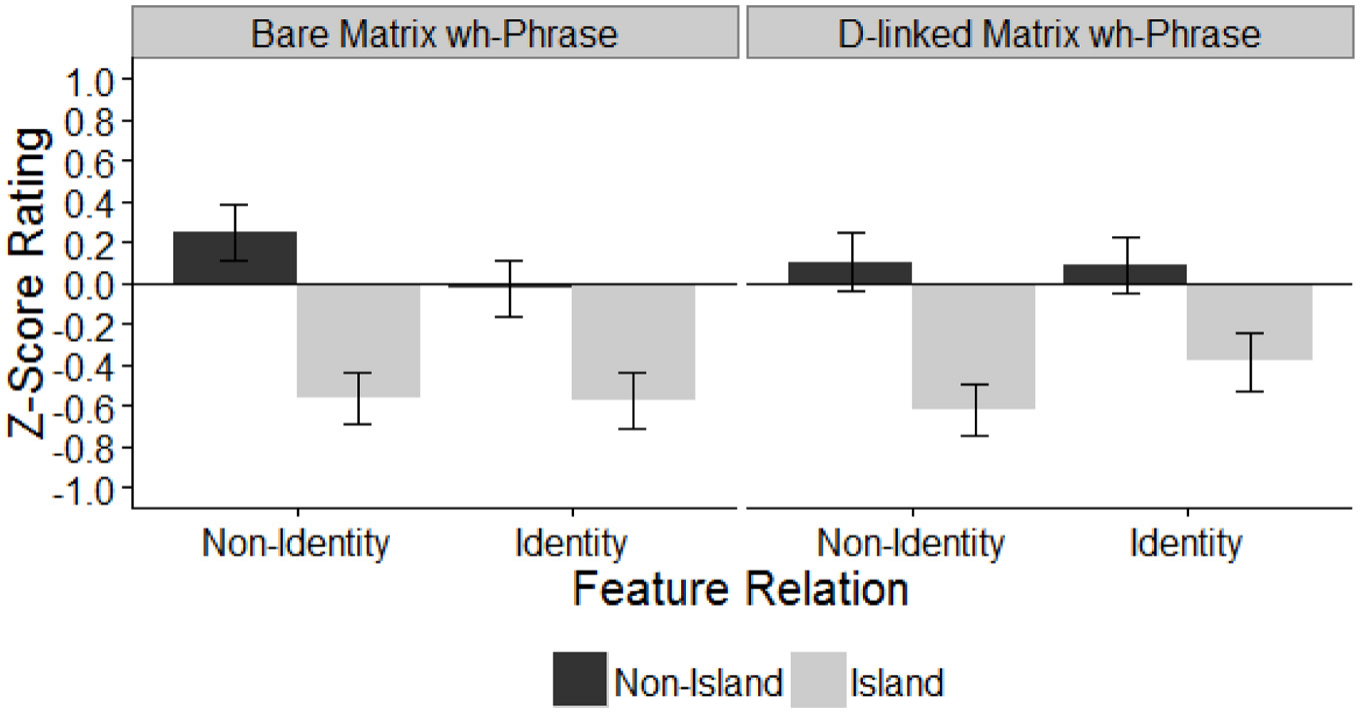
**Mean *z*-score acceptability rating in Experiment 2.** Error bars indicate ± 1 SE.

Similar to Experiment 1, all four island conditions were judged as less acceptable than their non-island counterparts (island mean *z*-score = -0.54, non-island mean *z*-score = 0.10), see **Figure 2**. Among the non-island conditions, the non-identity bare matrix *wh*-phrase condition received the highest rating (mean = 0.25), but we will leave this aside as it bears no relevance to our goal of testing the predictions of Featural RM. The other non-island conditions were judged similarly with mean *z*-score ratings around zero (means -0.03, 0.10, and 0.09). Among the island conditions, the D-linked identity condition was rated as the most acceptable (mean = -0.38). The remaining three extraction conditions received similar ratings (means -0.57, -0.58, and -0.62).

The Linear Mixed Effect model analysis confirmed that the overall pattern is consistent with Experiment 1. **Table 5** presents the estimated coefficients, the standard error, and the estimated *p*-value for the Linear Mixed Effect model with islandhood, feature relation, and matrix *wh*-phrase as fixed effects and random intercepts for participants and items.

**Table 5:** Fixed effects summary for Experiment 2 with by-participant and by-item random intercepts for islandhood, feature relation, and matrix *wh*-phrase type.

	Estimate	*SE*
Intercept	-0.22ˆ***	0.05
Islandhood	-0.64ˆ***	0.12
Feature relation	-0.02	0.06
Matrix *wh*-phrase	-0.02	0.05
Islandhood × Feature relation	0.26ˆ**	0.10
Islandhood × Matrix *wh*-phrase	-0.09	0.10
Feature relation × Matrix *wh*-phrase	-0.26ˆ**	0.10
Islandhood × Feature relation × Matrix *wh*-phrase	0.01	0.19

*The maximal random effects model did not converge; this model has random slopes for islandhood, feature relation, and their interaction.*

*^∗^p ≤ 0.05, ^∗∗^p ≤ 0.01, ^∗∗∗^p ≤ 0.001.*

As in Experiment 1, there was a main effect of islandhood, but there was no main effect of either feature relation or matrix *wh*-phrase. Importantly, there was an interaction of islandhood and feature relation as well as feature relation and matrix *wh*-phrase, which suggests that the feature relation factor modulates the effects of islandhood or matrix *wh*-phrase type on the acceptability. Planned pairwise comparisons among island conditions revealed no significant difference between the bare identity condition and the inclusion condition (β = 0.04, *SE* = 0.10, *p* > 0.1). This suggests that the D-linking amelioration effect was not observed for the inclusion configuration. Additionally, there was no significant difference between the inclusion and reverse inclusion conditions (β = 0.06, *SE* = 0.09, *p* > 0.1). On the other hand, the D-linked identity condition is significantly more acceptable than the inclusion condition (β = 0.23, *SE* = 0.11, *p* = 0.05), and marginally more acceptable than the bare identity condition (β = -0.19, *SE* = 0.11, *p* < 0.1). This pattern suggests that the D-linked identity condition showed a reliable amelioration of *wh*-island violations. As reverse inclusion patterns with inclusion, there is no significant difference between reverse inclusion and bare identity (β = -0.01, *SE* = 0.1, *p* > 0.1), but D-linked identity is marginally more acceptable than reverse inclusion (β = 0.18, *SE* = 0.1, *p* = 0.07).

### Discussion

Replicating the findings from Experiment 1, *wh*-island violations with D-linked identity received a reliably higher acceptability rating than bare identity or inclusion configurations. Furthermore, there was no clear evidence for amelioration of the *wh*-island violation in the inclusion condition. This selective *wh*-island amelioration effect is, again, not easily explained by Featural RM, which predicts that the inclusion configuration should be rated as more acceptable than bare or D-linked identity conditions. Finally, the finding that inclusion and reverse inclusion do not differ in acceptability also conflicts with the predictions of Featural RM.

The absence of an amelioration effect in the inclusion condition was surprising, given that amelioration effects in the inclusion configuration have been widely reported in the literature (Pesetsky, 1987; Cinque, 1990; Alexopoulou and Keller, 2013; Goodall, 2015). Experiment 3 explores whether the animacy of *wh*-phrases may play a role in amelioration of *wh*-island violations.

## Experiment 3

Experiment 2 provided no evidence for *wh*-island amelioration in the inclusion configuration. One plausible source of this unexpected finding is the number of animate nouns in the stimuli. Examples for *wh*-island amelioration in the literature typically included a single animate *wh*-phrase (5a), whereas the stimuli used in Experiment 2 (5b) included two animate *wh*-phrases.

(5) a. Which book did you persuade which person to read __? (Pesetsky, 1987)
      b. Which athlete did you wonder who would recruit __? (from **Table 3**)

It is plausible that having two animate *wh*-phrases makes them less distinct from one another, which may have increased confusability or processing demands in our stimuli. As discussed above, this is predicted by the similarity-based interference approach. In order to address this question, Experiment 3 replaces the animate *wh*-phrase [e.g., *which athlete* in (5b)] with an inanimate *wh*-phrase to more closely resemble the examples from the literature.

### Method

#### Participants

Thirty-one self-reported native English speakers participated via Amazon Mechanical Turk. They were paid $0.50 for completing the task.

#### Materials

The stimuli for this experiment consisted of 24 sets of biclausal sentences, following the same 2 × 2 × 2 design used in Experiment 2, with three factors: islandhood, feature relation, and matrix *wh*-phrase (see **Table 6**). The non-island conditions were identical to those in Experiment 2, where the matrix *wh*-phrase was animate. In the new island conditions, on the other hand, the fronted *wh*-phrase was changed from an animate to an inanimate noun (e.g., *which event*). Because the animacy of the fronted NP has changed, *what* replaces *who* as the bare matrix *wh*-word in the bare identity and reverse inclusion conditions (i.e., *What did you wonder…?)*.

**Table 6:** Sample item set from Experiment 3.

Bare matrix *wh*-phrase	Non-identity	Non-island (Animate)	Who __ wondered which family should host the event?
		Island (Inanimate)	What did you wonder which family should host __?
			*(Reverse Inclusion)*
	Identity	Non-island (Animate)	Who __ wondered who should host the event?
		Island (Inanimate)	What did you wonder who should host __?
			*(Bare Identity)*

D-linked matrix *wh*-phrase	Non-identity	Non-island (Animate)	Which graduate __ wondered who should host the event?
		Island (Inanimate)	Which event did you wonder who should host __?
			*(Inclusion)*
	Identity	Non-island (Animate)	Which graduate __ wondered which family should host the event?
		Island (Inanimate)	Which event did you wonder which family should host __?
			*(D-linked Identity)*

The 24 items were counter-balanced across eight lists, such that each participant saw only one version of each. Forty-eight filler items of comparable length and varying acceptability were randomly interspersed with these target items for a total of 72 items.

#### Procedure and Data Analysis

The procedure and data analysis method were identical to those of Experiment 2.

### Results

**FIGURE 3:**
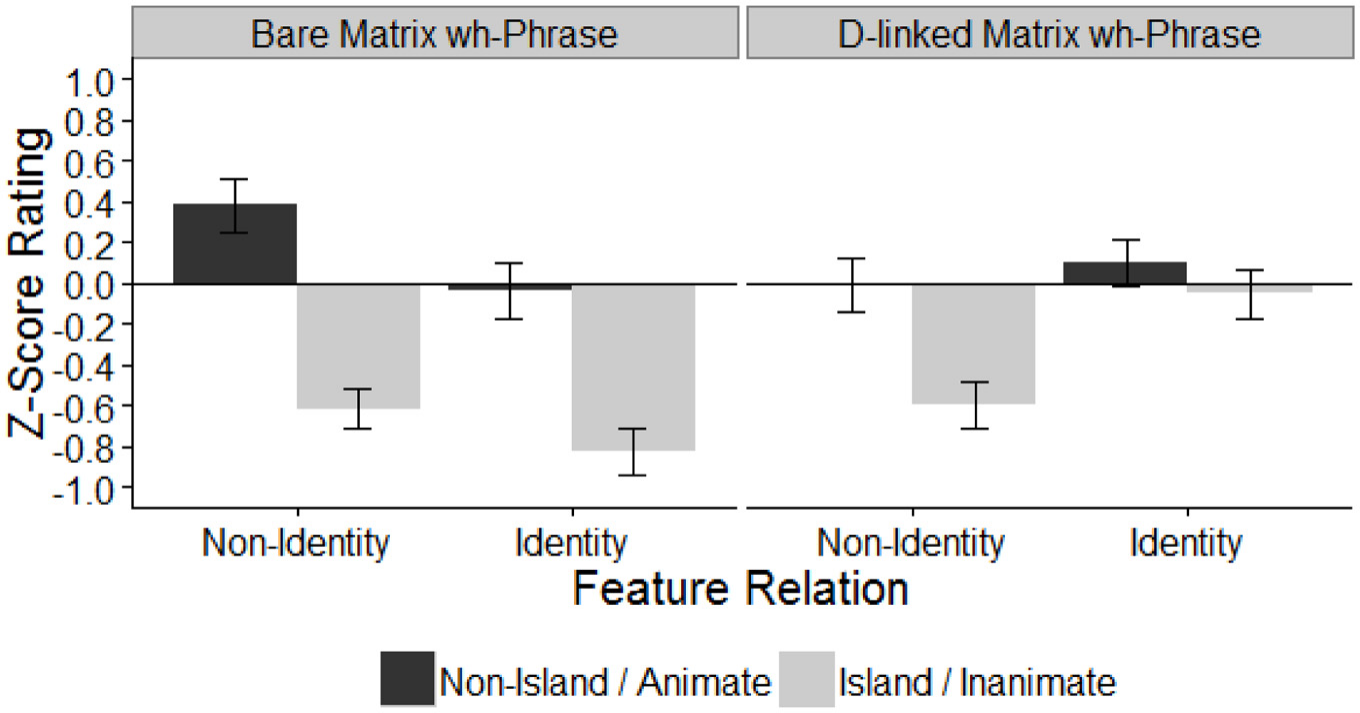
**Mean *z*-score acceptability rating in Experiment 3.** Error bars indicate ± 1 SE.

The acceptability judgment pattern in this experiment (**Figure 3**) resembles that of Experiment 2, as the D-linked identity condition received the highest rating among the extraction conditions (-0.06 vs. -0.62, -0.83, and -0.60).

These data were submitted to Linear Mixed Effect model analyses, which used islandhood, feature relation, and matrix *wh*-phrase as fixed effects and random intercepts for participants and items. The coefficient estimates, standard error, and estimated *p*-values are presented in **Table 7**.

**Table 7:** Fixed effects summary for Experiment 3 with by-participant and by-item random intercepts for extraction type, feature relation, and matrix *wh*-phrase type.

	Estimate	*SE*
Intercept	-0.21^∗∗∗^	0.04
Islandhood	-0.63^∗∗∗^	0.08
Feature relation	0.02	0.06
Matrix *wh*-phrase	0.14^∗∗^	0.05
Islandhood × Feature relation	0.31^∗∗^	0.11
Islandhood × Matrix *wh*-phrase	0.52^∗∗∗^	0.09
Feature relation × Matrix *wh*-phrase	0.62^∗∗∗^	0.09
Islandhood × Feature relation × Matrix *wh*-phrase	0.25	0.19

*The maximal random effects model did not converge; this model has random slopes for islandhood, feature relation, and their interaction.*

*^∗^p < 0.05, ^∗∗^p < 0.01, ^∗∗∗^p < 0.001.*

The results revealed the same main effect of islandhood as in the previous experiments due to the decreased acceptability of the island violating conditions (island mean = -0.52, non-island mean = 0.11). Also, all three of the pairwise interactions are significant: islandhood and feature relation, islandhood and matrix *wh*-phrase, and feature relation and matrix *wh*-phrase. This suggests that all of these factors influence acceptability, even though the three-way interaction is not significant.

Next, following the data analysis procedure in Experiment 2, planned pairwise comparisons of the island conditions were conducted in order to examine the precise distribution of the amelioration effect. Replicating the results of our previous experiments, the D-linked identity condition is significantly more acceptable than the inclusion condition (β = 0.54, *SE* = 0.09, *p* < 0.001) as well the bare identity condition (β = 0.78, *SE* = 0.12, *p* < 0.001). Also replicating Experiment 2, no difference was found between the inclusion and reverse inclusion conditions (β = 0.02, *SE* = 0.09, *p* > 0.1). Importantly, unlike Experiment 2, we found that the inclusion condition is significantly more acceptable than the bare identity condition (β = -0.23, *SE* = 0.09, *p* < 0.05). Again, reverse inclusion patterns with inclusion, so it is significantly more acceptable than bare identity (β = -0.21, *SE* = 0.09, *p* < 0.05) and marginally less acceptable than D-linked identity (β = 0.13, *SE* = 0.07, *p* = 0.07).

### Discussion

Once again, this experiment found that the D-linked identity condition was more acceptable than the other island conditions. Also, the reverse inclusion conditions patterned with the inclusion conditions. Unlike Experiment 2, however, we found evidence for *wh*-island amelioration in the inclusion configuration, as the inclusion island condition was judged as more acceptable than the bare identity island condition. The fact that this effect was only found in Experiment 3 could be taken to suggest that the animacy manipulation plays a critical role in its emergence.

However, there are reasons to be cautious of this interpretation. In Experiment 3, island and animacy factors were confounded as the fronted *wh*-phrases were always inanimate in the island conditions. This design does not allow a direct comparison of *wh*-island violations with fronted animate *wh*-phrases to those with inanimate ones. Experiment 4 explores this issue by manipulating animacy within the island conditions.

## Experiment 4

This experiment manipulates animacy and feature relation as in **Table 8**, in order to investigate whether *wh*-island amelioration in inclusion configurations is directly conditioned by the animacy of the fronted *wh*-phrase.

**Table 8:** Sample item set from Experiment 4.

Animate	Bare Identity	Who did you wonder who should host __?
	Inclusion	Which visitor did you wonder who should host __?

Inanimate	Bare Identity	What did you wonder who would host __?
	Inclusion	Which event did you wonder who should host __?

This allowed us to investigate the extent to which animacy contributed to wh-island amelioration effects. Given the results of Experiment 3, we predicted that the contrast between the inclusion and bare identity conditions should only appear in conditions with an inanimate *wh*-phrase.

### Method

#### Participants

Twenty-nine self-reported native English speakers participated via Amazon Mechanical Turk. They were paid $0.50 for completing the experiment. Three additional participants were excluded for using a single value (*n* = 1) or only the extremes of the scale (*n* = 2) during the calibration items.

#### Materials

The stimuli for this experiment consisted of 24 sets of biclausal sentences with a 2 × 2 design (**Table 8**), using animacy of the matrix *wh*-phrase (animate vs. inanimate) and feature relation (bare identity vs. inclusion) as factors. These items were largely based on stimuli from the previous experiments. The 24 test items were counter-balanced across four lists, such that each participant only rated a single item from each set. The addition of 48 length-matched filler sentences resulted in a total of 72 items.

#### Procedure and Data Analysis

The procedure and data analysis method were identical to those of previous experiments. Regardless of the presence of a significant interaction, planned pairwise comparisons of feature relation within animacy were conducted to directly test whether the amelioration effect of inclusion was modulated by animacy of the fronted *wh*-phrase.

### Results

**FIGURE 4:**
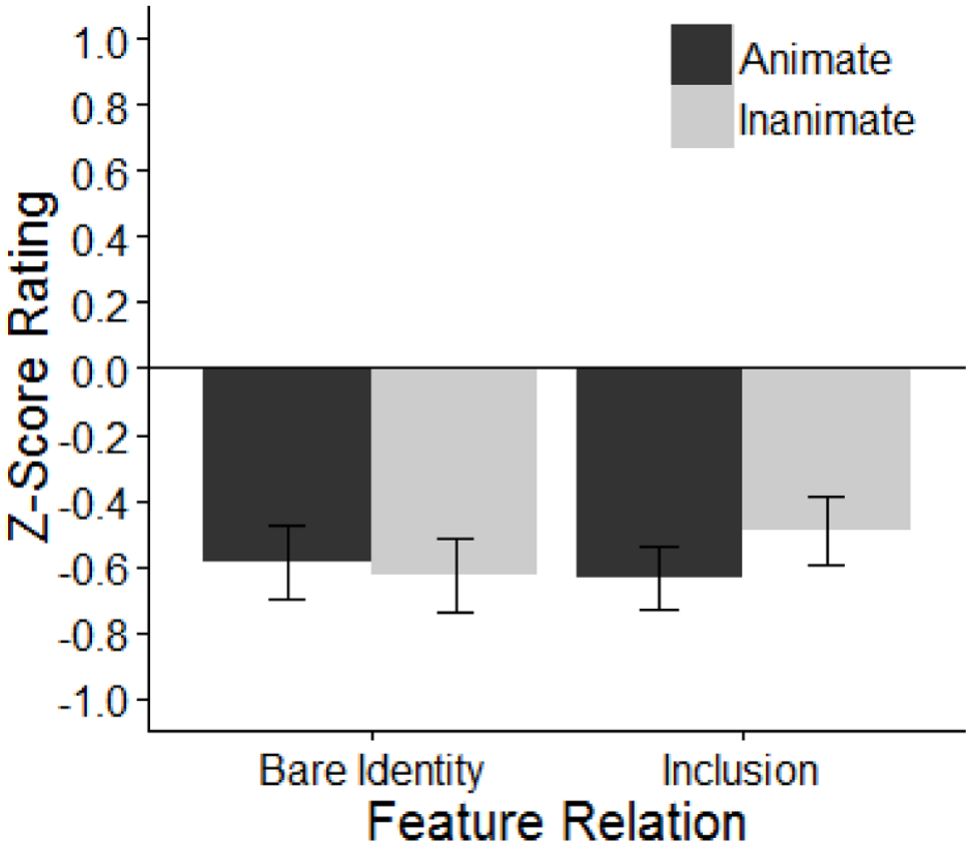
**Mean *z*-score acceptability rating in Experiment 4.** Error bars indicate ± 1 SE.

**Figure 4** presents the mean *z*-score ratings in each condition. Overall, inanimate *wh*-phrase conditions are rated as more acceptable than those with animate *wh*-phrases (inanimates = -0.55, animates = -0.61), but the bare identity and inclusion conditions show little difference in their acceptability ratings (bare identity = -0.59, inclusion = -0.57). Within the animate conditions, bare identity and inclusion show little difference in their acceptability ratings (-0.59 vs. -0.63). Within the inanimate conditions, however, inclusion was rated as more acceptable than bare identity (-0.51 vs. -0.60).

These data were analyzed using a Linear Mixed Effect model analysis with feature relation and animacy as fixed effects. The coefficient estimates, standard error and estimated *p*-values are given in **Table 9**.

**Table 9:** Fixed effects summary for Experiment 4 with by-participant and by-item random intercepts for feature relation and animacy of the matrix *wh*-phrase.

	Estimate	*SE*
Intercept	-0.59ˆ***	0.05
Feature relation	-0.05	0.06
Animacy	0.05	0.05
Feature relation Animacy	-0.18	0.11

*†p ≤ 0.1, ^∗^p ≤ 0.05, ^∗^p ≤ 0.01, ^∗∗∗^p ≤ 0.001.*

The model revealed no main effect of animacy or feature relation, but there was a marginal interaction between the two factors. Planned pairwise comparisons revealed that inclusion was marginally more acceptable than bare identity when the extracted *wh*-phrase was inanimate (inanimate: β = 0.13, *SE* = 0.07, *p* < 0.1), but not when the extracted phrase was animate (β = -0.04, *SE* = 0.07, *p* > 0.1).

### Discussion

This experiment investigated whether the animacy distinctness between two *wh*-phrases is a pre-requisite for *wh*-island amelioration in inclusion configurations. The results provide weak support for this hypothesis: when the fronted *wh*-phrase was animate, there was little difference between bare identity and inclusion conditions, but there was a marginal difference between these configurations when the fronted *wh*-phrase was inanimate. This finding has two implications. First, the results of Experiments 3 and 4 taken together suggest that the animacy of the extracted *wh*-phrases can modulate *wh*-island amelioration effects, but that the effect can be weak. Second, *wh*-island amelioration in inclusion configurations is generally not as robust as it has been reported in the literature; a weak amelioration may emerge when the fronted *wh*-phrase and intervener are distinct in animacy, but its effect is clearly not as consistently present as the amelioration effect observed in D-linked identity configuration in Experiments 1 through 3.

## General Discussion

The main goal of this study was to investigate the distribution of *wh*-island amelioration effects, and the extent to which they are modulated by morpho-syntactic and semantic features of *wh*-phrases. Specifically, we tested the acceptability of a *wh*-island violation involving two D-linked *wh*-phrases (i.e., D-linked identity) against violations with an intervening bare *wh*-phrase (i.e., inclusion) or with no D-linked *wh*-phrases (i.e., bare identity).

There are two main findings from the experiments reported above. First, we found consistent evidence against the predictions of Featural RM about D-linked identity configurations: such configurations reliably led to a higher acceptability than inclusion configurations. Featural RM predicts the opposite. Moreover, a study that was conducted in parallel in French used a similar design to our Experiment 3 and found the same pattern (Villata et al., in press). Thus, the increased acceptability of the D-linked identity configuration is robust across experiments and across English and French.

Second, we found that the D-linking amelioration effect for *wh*-island violations can be modulated by animacy, although the animacy effects were not always robust. Experiment 2 used only animate *wh*-phrases and found no evidence for *wh*-island amelioration in the inclusion configuration. Experiment 3 used inanimate nouns for extracted *wh*-phrases, and revealed evidence for amelioration in the inclusion configuration. This contrast between the experiments suggests that animacy might play a role. However, this effect did not hold robustly in Experiment 4, which showed that the amelioration effect was somewhat stronger for inclusion configuration than bare identity condition, which in turn showed no sign of amelioration regardless of the animacy manipulation. While a complete understanding of the role of animacy or the status of the inclusion configuration awaits further research, it is safe to conclude at this point that the *wh*-island amelioration effects for the inclusion configuration are not as robust as it has been reported in the literature.

These findings are summarized in (6), which depicts the ranking of acceptability variation among the *wh*-island violations that were examined in this paper. We will now discuss the theoretical implications of these findings.

(6) Bare identity ≤ (Reverse) inclusion with an animate *wh*-phrase extraction ≤ (Reverse) inclusion with an inanimate *wh*-phrase extraction < D-linked identity ≤ no extraction

### Implications for Featural RM

Our data suggests that Featural RM does not fully account for the distribution of *wh*-island amelioration effects, especially the fact that the D-linked identity configuration led to a robust amelioration effect. We do not present this as an argument against Featural RM *per se*, but minimally something else must be said to account for the behavior of D-linked *wh*-items beyond the inclusion/identity featural distinction. One potential implication is that the set of morpho-syntactic features assumed in papers by Rizzi and colleagues may need to be enriched. We will explore below the addition of Topic or Animacy features, but demonstrate that neither of these features provides a satisfactory explanation.

Rizzi (personal communication) suggests that the extracted D-linked *wh*-phrase has a [+Topic] feature that the intervening D-linked *wh*-phrase does not, as this feature is only licensed by the left periphery of the matrix clause (for a similar suggestion that the extracted *wh*-phrase may have a presupposition feature, see Grohmann, 2000; Boeckx and Jeong, 2003). If this is the case, then the sentences with two D-linked phrases are cases of inclusion rather than identity (7).

(7) **Which athlete** did you wonder **which coach** would recruit **__**?
      [+Q, +N, +Topic] [+Q, +N] [+Q, +N, +Topic]

This amendment allows Featural RM to account for the increased acceptability of the D-linked identity configuration. However, this featural augmentation does not explain why this configuration should be reliably more acceptable than the inclusion condition with a bare *wh*-phrase in the intervener position. Given the feature sets assumed in (7), both of these configurations are inclusion configurations, which are not predicted to show a contrast in acceptability. If we were to grade acceptability based on the degree of featural overlap, the prediction would again go the wrong direction: the bare inclusion condition should have less featural overlap, and therefore be more acceptable than the D-linked identity condition under the analysis in (7).

Another morpho-syntactic feature that may deserve to be added to the Featural RM framework is an animacy feature. It is typically assumed that animacy features do not actively participate in syntactic operations in English. However, animacy is known to play important roles in syntax of other languages (e.g., Slavic languages, see Rappaport, 2003). Our observations of superior *wh*-island amelioration effects for inanimate *wh*-phrases may be the first evidence that animacy plays an important role in English syntax as well. However, the addition of an animacy feature with the same status as e.g., [+Q] above is not fully motivated by our data either. First, it offers no explanation for the observed acceptability contrast between the D-linked identity and inclusion configuration in Experiments 1 and 2. Second, using animacy features in Experiment 3 would change the D-linked identity feature relation to that of a reverse inclusion, as shown in (8). Under this configuration, Featural RM predicts the sentence to be equally as degraded as identity configurations, which is the opposite of what was found in Experiment 3. Rather, if Experiment 3 is taken at face value, (8) should be ameliorated simply because the two D-linked *wh*-phrases have a different value for animacy.

(8) **Which award** did you wonder **which actress** should receive **__**?
      [+Q, +N] [+Q, +N, +animate] [+Q, +N]

Finally, incorporating an animacy feature would predict that animacy based amelioration effects hold robustly across all *wh*-island violations, but this prediction is inconsistent with the observation in Experiment 4 that the animacy manipulation showed a selective, weak modulation of the acceptability of the inclusion conditions but not the bare identity configuration. While an animacy distinction is clearly relevant, it cannot easily be captured in featural terms.

In summary, it is not obvious what featural adjustments could account for the amelioration patterns we have shown in this paper in a way that is entirely internal to the principles of Featural RM.^4^ If this effect cannot be accounted for with featural manipulations, then (minimally) something external to the featural system must lead to the amelioration pattern.

### Memory Constraints and Semantic Distinctness in Acceptability Variation

More generally, these results present a challenge to any account of *wh*-island effects that assumes that D-linked identity examples are acceptable or fully amelioriated: the variable amelioration effect for even this case suggests that some constraint like Relativized Minimality may well be active (in contrast to accounts of D-linking that simply assign it a different LF where the constraint leading to the violation is not at play; Pesetsky, 1987, 2000 on superiority). An explanation for the distribution of *wh*-island amelioration effects in our experiments must take into account the superior amelioration effects in D-linked identity configurations, as well as the fact that extraction of an inanimate *wh*-phrase sometimes leads to a further increase in acceptability. Before we present such explanations, we first argue for a new descriptive generalization: the degree of semantic distinctness of the extracted *wh*-phrase and the intervener (rather than the distinctness of morpho-syntactic features) predicts the distribution of *wh*-island amelioration effects.

We suggest that participants in these experiments were able, to varying degrees, to use *semantic distinctness*, rather than morphosyntactic distinctness, as a strategy for interpreting ill-formed *wh*-island examples. First, we will adopt a broadly Hamblin semantics of *wh*-questions, and assume that (i) questions denote a set of possible answers (Hamblin, 1973; see also Karttunen, 1977, and many others), and (ii) *wh*-phrases denote a set of potential referents (Hamblin, 1973; Kratzer and Shimoyama, 2002). Intuitively, the set of referents for the *wh*-item in a single-*wh* question corresponds to possible fragment NP answers to that question. Under this family of assumptions, bare *wh*-phrases like *who* denote the set of all human individuals, whereas a D-linked *wh*-phrase like *which award* would denote a presupposed set of entities satisfying the NP restrictor, in this case award, and require the answer to the *wh*-question to be constructed from some referent in this set only. With these assumptions, let us examine the distinctness of sets of individuals or objects denoted by *wh*-phrases in **Table 10**, which illustrates the main feature configurations that were investigated in our acceptability judgment experiments.

**Table 10:** Distribution of amelioration effects and semantic distinctness.

Conditions	Sentence	Amelioration?	Semantic distinctness
Bare identity	Who/what did you wonder who would host__?	No	Non-distinct
Inclusion (animate)	Which visitor did you wonder who would host __?	No	Non-distinct
Inclusion (inanimate)	Which event did you wonder who would host __?	Maybe?	Distinct
D-linked identity	Which visitor did you wonder which family would host __?	Yes	Distinct
D-linked identity	Which event did you wonder which family would host __?	Yes	Distinct

In the bare identity condition with *who* as an extracted *wh*-phrase, both the extracted *wh*-phrase and the intervener denote the set of all humans, and therefore their domains are identical and non-distinct. If the extracted *wh*-phrase is *what*, we assume that *what* denotes a set of everything in the world, which includes human individuals.^5^ Here, the set denoted by *what* is a superset of the set denoted by *who*, and these sets are thus overlapping. As for the inclusion configuration with animate *wh*-phrases, *which visitor* denotes a presupposed set of visitors, while *who* denotes a set of all human individuals. Thus, the sets of individuals denoted by these two *wh*-phrases are also overlapping. On the other hand, for the inclusion configuration with inanimate and animate *wh*-phrases, the set denoted by *which event* and the set denoted by *who* are distinct. This explains the amelioration effect that was observed in the comparison of Experiments 2 and 3. Finally, in the D-linked identity conditions, the sets of individuals or objects denoted by the two *wh*-phrases (*which visitor* and *which family*, or *which event* and *which family*) are clearly distinct. Thus, these observations lead to the generalization that the *wh*-island violations that were amenable to amelioration effects were those in which the sets denoted by the extracted *wh*-phrase and the intervener are distinct. We take this as a necessary condition for *wh*-island amelioration.

The semantic distinctness of the *wh*-phrases provides the beginnings of an explanation of many of the patterns in our data, but clearly we do not have evidence for any sort of categorical amelioration; in fact, our results could be taken as evidence against it. One possible explanation for this state of affairs is that similarity-based interference during memory retrieval operations is sensitive to the semantic distinctness of two *wh*-phrases. As noted in the Introduction, it has been widely observed that the processing of filler-gap dependencies can be impeded when the dependencies contain two similar NPs. This similarity interference effect is considered to follow from limitations of the memory system in either encoding two similar NPs as distinct items, or in retrieving the target NPs with accurate syntactic and semantic features. It is plausible that the semantic distinctness of *wh*-phrases modulates the ease of encoding or retrieval processes, and when these processes are readily performed, participants may perceive the *wh*-island violations to be less severely degraded. In this sense, the semantic distinctness of *wh*-phrases may serve as a formal characterization of NPs that are particularly confusable for memory operations.

This psycholinguistic explanation for the role of semantic distinctness and memory constraints has implications for theories of islands and syntactic amelioration effects in general. We suggest two potential approaches for integrating syntactic and psycholinguistic constraints, both of which are equally compatible with our findings. The first approach is to reduce island constraints to cognitive constraints on memory operations, such that “island violations” merely reflect difficulties in establishing *wh*-dependencies during real-time parsing (Kluender and Kutas, 1993; Hofmeister and Sag, 2010; for related explanations for Superiority effects, see Hofmeister et al., 2013). With respect to *wh*-islands, according to this reductionist approach, what used to be considered violations of Featural RM constraints would be reanalyzed as severe instances of similarity-based interference effects, which are sensitive to both syntactic and semantic features of retrieval candidates. Simplifying the theory of grammar and postulating fewer constraints that are specific to linguistic representations is a welcome result (Chomsky, 1995; Phillips, 2013), and it highlights how syntactic theories can be refined by a further collaboration between linguistics and broader cognitive science research. The future agenda for this approach includes extension of experimental investigations to other syntactic phenomena that Featural RM provided explanations for (e.g., intervention effects in *combien* extraction in French; Obenauer, 1983, 1994), as well as addressing counter-arguments for cognitive explanations of island constraints (Sprouse et al., 2012; see also Phillips, 2006). We leave these questions for future research.

The second approach for integrating syntactic constraints on *wh*-dependency formation and memory constraints is to situate similarity interference effects in *repair processes* that the parser initiates in order to cope with a violation of formal, syntactic constraints; we term this approach the Amelioration-as-Repair hypothesis. This explanation of amelioration effects relies on the following three assumptions. First, we assume that acceptability judgment intuitions minimally reflect the well-formedness of syntactic derivations and semantic representations that the parser assigns to a given sentence. When this process fails due to linguistic or other cognitive constraints, we perceive degradation in sentence acceptability (Schütze, 1996), and the severity of degradation reflects the number of constraint violations at all levels of representations (Legendre et al., 1991; Keller, 2000; Smolensky and Legendre, 2006; Haegeman et al., 2014). Second, we also assume that syntactic constraints on *wh*-islands do play an important role in accounting for the general acceptability degradation due to extraction out of *wh*-islands, and this constraint could be the original Relativized Minimality constraint in Rizzi (1990, 2004) which did not distinguish bare identity *wh*-island from inclusion *wh*-island. Finally, we also assume that in the face of sentences that violate syntactic constraints, the parser attempts to repair the structure in order to assign an interpretation to the structurally unintegrated *wh*-phrase. Such interpretive repair processes are well documented in the psycholinguistics literature on severe garden-path sentences (e.g., Christianson et al., 2001; Ferreira and Patson, 2007). While this style of repair may not “cancel” the initial violation of syntactic constraints, it would at least provide a strategy for obtaining a legitimate semantic representation for the sentence that can be passed onto the interpretive process.

Given these assumptions, acceptability judgment data should reflect the degree to which this repair process is able to (a) identify a gap position inside an island, and (b) retrieve the relevant *wh*-phrase in order to complete the *wh*-dependency for the semantic representation. Under the Amelioration-as-Repair approach, it is during this repair/retrieval process that the similarity interference effects arise. It is well known that the parser typically respects island constraints during real-time sentence processing (e.g., Stowe, 1986; Traxler and Pickering, 1996); thus, initially the parser should generate an ungrammatical structure with no gap for the *wh*-phrase. This syntactic violation initiates the repair process, and the search for a gap inside an island. This search process identifies a verb with a missing complement, which indicates that the verb could be a host for the gap. This gap identification subsequently triggers a retrieval of a *wh*-phrase, using the thematic role and morphological features as retrieval cues.^6^ This retrieval process should be sensitive to the semantic distinctness of *wh*-phrases. If the repair process fails due to similarity interference effects (e.g., in the bare identity condition), the semantic representation would veridically reflect the syntactic violation of the *wh*-island constraint (i.e., no gap for the *wh*-phrase), and the sum of these two violations results in more severe degradation. On the other hand, if the parser identifies a gap inside an island due to the lack of similarity interference effects (e.g., in D-linked identity conditions with semantically distinct *wh*-phrases), the resulting semantic representation no longer contains any violation, even though it is derived from a structure that does, and therefore the only source of acceptability degradation is the initial violation of the *wh*-island constraint (see Huang, 1982 for arguments that the semantic representation of islands with argument gaps does not incur any violation).

One consequence of the Amelioration-as-Repair hypothesis is that it provides a new direction toward a mechanistic understanding of acceptability judgment in general. To this day, even though acceptability judgment data has served as the primary source of data for linguists, there is very little theory of how such intuitions arise (cf. Schütze, 1996), or how the process of judging sentence acceptability reflects psycholinguistic constraints. As such, regardless of whether island constraints or Featural RM should remain as a formal constraint on linguistic representations, integration of perspectives and insights from psycholinguistics could help advance the field of syntax.

Finally, we note that either approach raises new research questions that need to be addressed in future research. First, the current study does not provide time course measures that shed light on the memory encoding and retrieval mechanisms that are assumed under either explanation. Second, it remains to be answered why the animacy-based modulation of *wh*-island amelioration effects was not reliably observed across experiments. Following the psycholinguistic explanations above, we tentatively suggest that the real-time encoding and comparison of semantic distinctness information could be subject to a variety of conceptual or cognitive factors that will then impact the behavior of amelioration. For example, accessing the set of all individuals denoted by *who* may be inherently complex when it is presented out of context, as in the current experiments. This difficulty may sometimes mask the potential advantage of semantic distinctness in the inclusion configuration with an inanimate *wh*-phrase, suggesting also that it may not be generally safe to test amelioration effects out of context.

## Conclusion

The present study investigated the distribution of *wh*-island amelioration effects, with a special focus on how it is modulated by morpho-syntactic features and semantic features of *wh*-phrases. We found that morpho-syntactic features alone, such as those to which Featural RM in its current form appeals, failed to account for the distribution of *wh*-island amelioration effects. We suggested that a full explanation of our results requires the consideration of semantic representations, which may, in turn, be related to constraints on the sentence processing mechanisms that give rise to similarity interference effects. This observation calls for future work that re-examines amelioration effects in other syntactic environments in light of constraints on sentence processing mechanisms.

## Conflict of Interest Statement

The authors declare that the research was conducted in the absence of any commercial or financial relationships that could be construed as a potential conflict of interest. The reviewer, Dario Leander Jim Felix Paape, and handling editor declared their shared affiliation, and the handling editor states that the process nevertheless met the standards of a fair and objective review.

## Footnotes

1 For a related study in French, see Villata et al. (in press).

2 While the present study does not directly tap the real-time generation of acceptability intuition, many studies have shown a correspondence between real-time comprehension difficulties and oﬄine judgment data in processing of filler-gap dependencies (see, for example, Gibson and Thomas, 1999; Hofmeister and Sag, 2010; Vasishth et al., 2010; Hofmeister et al., 2013).

3 The overall pattern in our results did not change when the analysis included participants that would be removed according to this criterion. In this paper, we only present data that excluded those participants, as we think that this exclusion increases the chance of veridically representing the acceptability contrasts between conditions.

4 One reviewer suggested the inclusion of both a topic and an animacy feature. In example (8), this would result in a configuration known as *intersection*, where the fronted *wh*-phrase and the *wh*-intervenor have distinct sets of features that share a subset (in the terms of **Table 1**, the fronted phrase is [+A,+B] and the intervener is [+A,+C]) (Belletti et al., 2012). However, it is unclear whether this configuration should pattern with disjunction or with intersection in acceptability judgments. Additionally, this does not address the concern that example (7) becomes a case of intersection with the addition of these features. It is still unclear why sentences like (7) are consistenly more acceptable than the other cases of inclusion included in our experiments.

5 There are three empirical reasons for assuming that *what* is underspecified for human or animacy features, and therefore is able to denote humans (see Grosu, 2003). First, *what* can be combined with either animate or inanimate nouns to form complex *wh*-phrases (e.g., *What doctor did you see? What textbook did you buy?*), whereas this type of composition is not possible for *wh*-phrases like *who* with clear human and animacy feature specification (^∗^*who doctor*). Second, the answer to *what* can be human or non-human, especially when there are multiple answers (e.g., *What can you see? John, Mary, and a tree.*). This is not possible for *wh*-phrases that are specified for human features (e.g., *Who did you see? ^∗^John, Mary, and a tree.*). Third, free relative clauses with *what* can take a human or a non-human referent (Grosu, 2003). In a sentence like *What I thought was a policeman was just a log*, the *wh*-phrase *what* is treated as human (a policeman) internally to the free relative clause, whereas it is treated as inanimate (a log) externally.

6 It is also plausible that the animacy effect observed in our experiments reflects the fit of the verb semantic retrieval cues and the *wh*-phrases (e.g., *event* may be a better object for *host* than *visitor*). Testing this hypothesis requires a careful control of verb-noun co-occurrence frequency and plausibility. We leave this question open for future research.
